# Associations of Nutritional Status with Full Immunization Coverage and Safe Hygiene Practices among Thai Children Aged 12–59 Months

**DOI:** 10.3390/nu14010034

**Published:** 2021-12-23

**Authors:** Chisa Shinsugi, Ann Mizumoto

**Affiliations:** 1 Department of Nutritional Epidemiology and Shokuiku, National Institute of Health and Nutrition, National Institutes of Biomedical Innovation, Health and Nutrition, Tokyo 162-8636, Japan; 2 Independent Researcher, Singapore 436932, Singapore; annmizumoto@gmail.com

**Keywords:** malnutrition, infants and young children, immunization, safe hygiene practices, primary health care, universal health coverage, Thailand

## Abstract

Prevailing prevention measures against morbidity, such as vaccination and safe hygiene practices, vary among local cultural contexts, and little is known about the extent to which these behaviors mitigate poor nutritional status in young children in Southeast Asia. We examined the associations between nutrition status with full immunization coverage, and water, sanitation and hygiene status among children aged 12–59 months in the 2015–2016 Thailand Multiple Indicator Cluster Survey (n = 9060). When adjusted for confounding factors, children with incomplete immunization status were more likely to be stunted (adjusted odds ratio (aOR) 1.47; 95% confidence interval (CI): 1.24–1.75, *p* < 0.001), wasted (aOR 1.67, 95% CI: 1.31–2.12, *p* < 0.001), and overweight (aOR 1.24, 95% CI: 1.01–1.51, *p* < 0.05), whereas children who used unimproved water sources were more likely to be overweight (aOR 2.43, 95% CI: 1.27–4.64, *p* < 0.01). The further implementation of simple and cost-effective health promotion activities and practices at the household level may be important interventions for healthy child growth and development, particularly under restricted living conditions due to COVID-19.

## 1. Introduction

Malnutrition in all its forms remains a major global challenge among infants and young children in low- and middle-income countries (LMICs) [1]. The importance of nutrition in early life for brain development [2] and long-term health risks, including obesity and diabetes [3], is widely acknowledged and urgently needs to be addressed. Various contributing factors such as health status and water, sanitation, and hygiene (WASH) are estimated to play a critical role in the improvement in the nutritional status among some African and South Asian countries [4]. These multisectoral efforts are required to address the increasingly complex challenges of alleviating malnutrition.

Comprehensive primary health care (PHC) strategies were emphasized to protect basic human rights, address the triple burden of malnutrition and, ultimately, achieve universal health coverage (UHC) [1,5]. Prevention-oriented health and WASH activities in PHC are becoming increasingly important, particularly under the current infection prevention and control measures to prevent the spread of coronavirus disease 2019 (COVID-19). Essential childhood immunizations such as the measles vaccine remain inadequate to fully protect children at risk of preventable deaths [6]. How nutrition-sensitive approaches contribute to improving nutritional status varies by context and more research is required [4]. A study from 35 LMICs, mostly African countries, suggested that unimproved sanitation, unsafe feces disposal, and partial vaccination status were relatively strongly associated with child stunting based on 20 factors from the UNICEF conceptual framework [7,8]. Socioeconomic status, which comprises household characteristics such as drinking water source and type of toilet facility, is associated with child stunting in rural communities, regardless of food security conditions [9]. One report showed that handwashing with soap and water before meals practiced by caregivers was protective against stunting among children aged 0–23 months in rural India, even without access to piped water [10]. A systematic review suggested that the improvement in household water quality contributed to a recovery from severe acute malnutrition [11]. UHC indicators including “3.b.1 Proportion of the target population covered by all vaccines included in their national program” under “Goal 03. Good Health and Well-Being”, as well as “6.1.1 Proportion of population using safely managed drinking water services” and “6.2.1 Proportion of population using safely managed sanitation services, including a hand-washing facility with soap and water” under “Goal 06. Clean Water and Sanitation”, are considered key factors in achieving health-related Sustainable Development Goals [12]. Greater attention towards the relationship between PHC and nutritional status is required because of important emerging evidence concerning the indirect role of PHC interventions in nutrition-sensitive programs. Studies investigating this association among infants and young children in Southeast Asia, however, are scarce.

In Thailand, the percentage of stunting and wasting among children under age 5 years is 10.5% and 5.4%, respectively, whereas the percentage of overweight children in this group is 8.2% [13]. The previous study in 35 LMICs found that preventive behaviors in PHC, such as immunization and WASH, including the safe disposal of child feces, were associated with nutritional status [7]. It is unclear whether this relationship can be generalized to Thailand. Moreover, little is known about the relationship between child malnutrition and lifestyle habits and household practices such as handwashing. This study aimed to examine the associations between nutritional status and PHC, including household sanitation, hygiene practices, and full vaccination coverage among Thai children aged 12–59 months.

## 2. Materials and Methods

### 2.1. Study Design and Study Sample

We used data extracted from the 2015–2016 Thailand Multiple Indicator Cluster Survey, which was conducted from November 2015 to March 2016. A multistage, stratified, random cluster sampling design was used to select the survey sample. A specified number of census enumeration areas (clusters) were systematically selected using probability proportional to size. After listing all households within a selected cluster, 20 households in each cluster were randomly selected, forming a total sample of 31,010 households. In the household questionnaire, 12,313 children under 5 years of age were included. The detailed survey design and methods were described in the final survey report [13]. The percentage of all children under age 5 years whose birth was registered with civil authorities was 99.5%. Of the eligible children under 5 years old (n = 12,313), 12,250 mothers and caregivers were interviewed (response rate 99.5%). After excluding biologically implausible values and missing values for outcome variables (n = 1175), as well as children under 12 months of age, a total of 9830 children were included in this analysis. Ultimately, a weighted sample of 9060 children (4661 boys and 4399 girls) was used.

### 2.2. Outcome Variables

Outcome variables were nutritional status, i.e., stunting, wasting, and overweight. Stunting was defined as length/height-for-age z-score < −2 standard deviations (SDs), with reference to World Health Organization growth standards [14]. Wasting was defined as weight-for-length/height z-score < −2 SD, and overweight was classified as WHZ > 2 SD.

### 2.3. Exposure Variables

We selected full immunization coverage and WASH practices in PHC and UHC as the dependent variables. Full immunization coverage was defined as a child having received all 12 vaccinations recommended in the national immunization schedule by their first birthday: Bacillus Calmette–Guérin (BCG), Polio1-Polio3, diphtheria–pertussis–tetanus (DPT1-DPT3), hepatitis B (HepB0-HepB3), and measles, mumps, and rubella (MMR1).

WASH practices were assessed using three indicators: use of improved drinking water sources, safe disposal of child feces, and having a location for handwashing. The indicator use of improved drinking water sources was classified as using any of the following types of water supply: piped water (into the dwelling, compound, yard or plot, to a neighbor, public tap/standpipe), tube well/borehole, protected well, protected spring, and rainwater collection. Bottled water was considered an improved water source only if the household was using it for handwashing and cooking. The indicator for safe disposal of child feces was defined as children whose last stools were disposed of safely (using a toilet or by rinsing the stool into a toilet or latrine). The indicator for handwashing location was defined as households with a specific place for handwashing with water and soap, or another cleansing agent.

### 2.4. Covariates

Birth weight (low birth weight < 2500 g or ≥2500 g), residential area (urban or rural), region (Bangkok, Central, North, Northeast, or South), household wealth index quintile (poorest, second, middle, fourth, or wealthiest), and mother’s education (none, primary, secondary, higher, missing or don’t know) were considered as covariates. The wealth index was constructed with principal component analysis using factors related to household wealth, such as ownership of consumer goods and dwelling characteristics [13].

### 2.5. Statistical Analysis

We analyzed the distribution of individual growth to determine the nutritional status of the study population. We then performed multiple logistic regression analyses to examine the association of nutritional status with full vaccination coverage and WASH status after adjustment for low birth weight, residential area, region, household wealth index quintile, and mother’s education, by child sex [15]. Adjusted odds ratios (aOR) with 95% confidence intervals (CI) were calculated, with a significance level of *p* < 0.05. Statistical analyses were performed using Stata Version 16.1 (StataCorp, College Station, TX, USA).

## 3. Results

**Figure 1:**
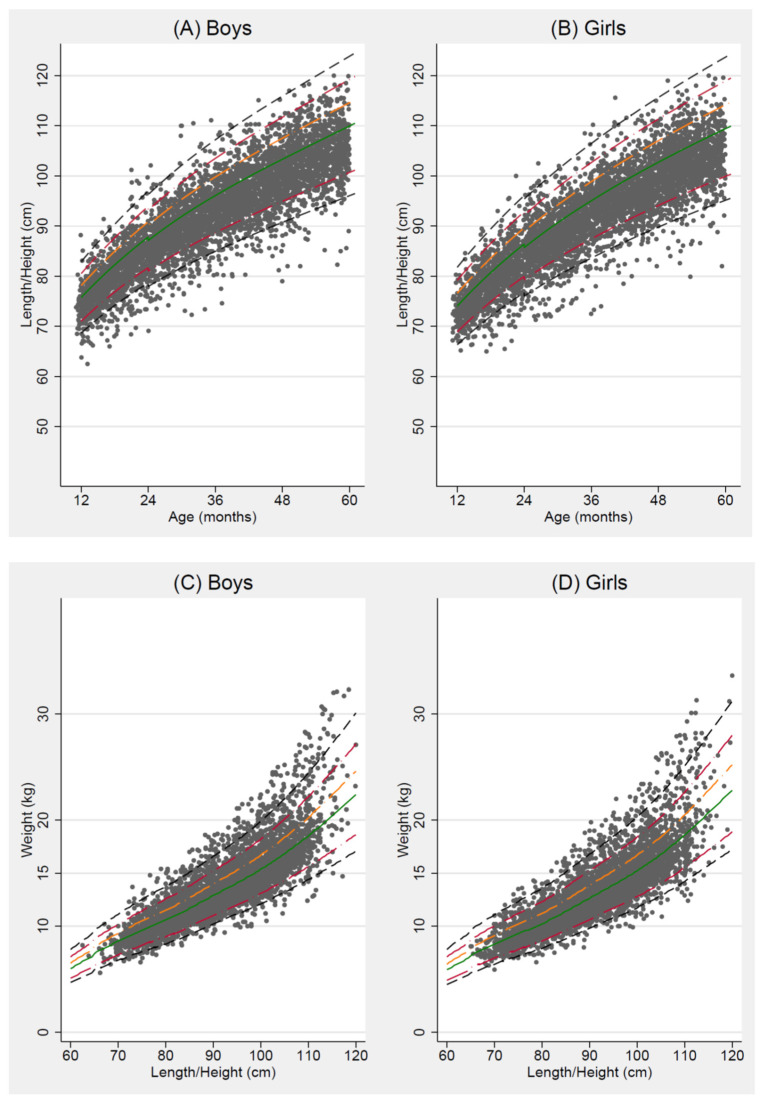
Distribution of length/height-for-age (HFA) for (**A**) boys and (**B**) girls, and weight-for-height (WFH) for (**C**) boys and (**D**) girls, from 12 to 59 months. The five lines represent the median (solid green line) and standard deviation (SD) from the median HFA and WFH according to World Health Organization Child Growth Standards: ±3 SD (dashed black line), ±2 SD (long-dashed cranberry line), +1 SD (long-dashed orange line).

Sex-disaggregated distributions of length/height-for-age and weight-for-height of children aged 12–59 months are presented in Figure 1. Children were concentrated in the range of −2 SD to +1 SD for length/height-for-age, regardless of their age in months.

The characteristics of study participants by sex are presented in Table 1. The mean age (±SD) of the included children was 35.5 ± 13.7 months. The percentage of concurrent stunting and wasting was 0.6%, and the percentage of concurrent stunting and overweight children was 1.2%. Most children were fully vaccinated (84.8%) and almost all included children had a vaccination card (98.4%). As for WASH indicators, most children had used improved water sources (86.2% of boys and 87.6% of girls). Only one in four children demonstrated the safe disposal of child feces (25.6% of boys and 26.2% of girls), whereas more than half of the households had a specific place for handwashing with water and soap or other cleansing agents (58.2% of boys and 60.4% of girls).

Table 2 shows the results of multiple logistic regression analyses for the associations of nutritional status with full vaccination and WASH status. When adjusted for confounding factors, children with incomplete vaccination coverage were more likely to be stunted (aOR 1.47, 95% CI: 1.24 to 1.75, *p* < 0.001), wasted (aOR 1.67, 95% CI: 1.31 to 2.12, *p* < 0.001), and overweight (aOR 1.24, 95% CI: 1.01 to 1.51, *p* < 0.05), whereas children who used unimproved water sources were more likely to be overweight (aOR 2.43, 95% CI: 1.27 to 4.64, *p* < 0.01).

## 4. Discussion

In the present nationally representative sample of Thai children aged 12–59 months, we examined the associations among immunization status, WASH status, and nutritional status. We found that children with incomplete vaccination coverage were more likely to be stunted, wasted, and overweight, whereas children who used unimproved water sources were more likely to be overweight.

Although the concurrence of stunting and overweight children was slightly observed, approximately 1 in 10 children were stunted or overweight in this study alone. Children with severe stunting may take longer to return to a healthy growth trajectory, so it is imperative to identify these children early and start immediate treatment. Many young children in LMICs are overlooked because they may not have their growth status regularly monitored during and after routine health checkups or immunization visits. Further studies are required to track the growth trajectory of young children and explore those factors that contribute to recovery from malnutrition.

In this study, children who failed to receive all twelve vaccinations before age 1, as recommended by the national immunization program, showed an association with malnutrition (stunting and overweight in boys and wasting in girls) in early life compared with children who were fully vaccinated by their first birthday. This result is consistent with previous studies reporting that children aged 6–23 months in South Asian countries who were partially vaccinated, defined as having received eight vaccinations (measles, BCG, DPT3, and Polio 3), were more likely to be severely stunted than fully vaccinated children [16]. However, the coverage for vaccines that require multiple doses tends to decline gradually. It is important to improve UHC, including strengthening vaccination programs in communities as a nutrition-sensitive intervention during the first 1000 days of life. Our findings suggest that such basic health services may also have a protective effect against malnutrition.

After adjustment for confounding factors, children in this study who lived in environments with poor hygiene practices (use of unimproved water sources in boys and unsafe disposal of child feces in girls) were more likely to be malnourished (overweight in boys and stunting in girls) compared with children who had appropriate hygiene practices. One possible explanation for the tendency for boys to be overweight in households with unimproved water sources is that, since eating out at street stalls is common in Thailand, their parents may be buying more sugar-sweetened beverages and other juices that young children prefer instead of drinking unsafe water at home. Moreover, the relationship between poor hygiene practices and malnutrition in this study is consistent with those of a previous study from 34 countries, which reported that child feces disposal in an improved toilet was negatively associated with stunting among children under the age of 5 [17]. In Thailand, over 40% of children aged 0–2 years have their diapers discarded into the garbage, which is classified as inappropriate disposal due to concerns of environmental hygiene [13]. While a systematic review found that the effects of WASH interventions alone on child growth were limited [18], multisectoral interventions that include WASH were not sufficiently studied in Southeast Asia. Promoting safe personal and community hygiene and sanitation practices in an environmentally sustainable manner, such as the encouragement of appropriate waste segregation, may help protect children’s growth and development. Further in-depth qualitative research is required, taking into account local context and traditional lifestyles, thus supporting the more effective implementation of nutrition-sensitive programs.

The present study has several limitations. First, causal inference cannot be asserted from the results due to the nature of the cross-sectional study design, except for the effect of full vaccination status before the first birthday on nutritional status among children aged 12–59 months. Second, we cannot completely rule out the potential effects of confounding factors, such as residual and unmeasured variables, despite adjusting for the main indirect factors, such as socioeconomic status. Since wealth index and mother’s education, covariates in this study, were associated with nutritional status, it would be useful to include socioeconomic status. Although these limitations must be considered when interpreting the results, this study revealed important information regarding the associations of nutritional status with full vaccination status and hygiene practices using nationally representative data of young children in Thailand. It is expected that the current COVID-19 pandemic will worsen the status of child nutrition and mortality in LMICs [19,20]. Restricted social activities together with limited resources, including financial and material support, may hamper the implementation of nutrition-specific programs, since health practitioners may need to focus on infection prevention and control measures. As the Tokyo Nutrition for Growth Summit 2021 has highlighted, integrating nutrition into UHC, despite the current resource-constrained environment of the COVID-19 pandemic [21], may be an effective multisectoral approach to improve nutritional status in early childhood.

## 5. Conclusions

This study showed that infants and young children in Thailand with incomplete vaccination coverage before their first birthday were associated with malnutrition (stunting and overweight in boys; wasting in girls). The study also found that children who lived in environments with poor hygiene and unsafe sanitation (use of unimproved water sources in boys and unsafe disposal of child feces in girls) were more likely to be malnourished (overweight in boys and stunting in girls). Our findings suggest that health promotion activities such as routine immunizations, as well as safe hygiene practices at the household level, are critical for healthy child growth. Further research is warranted to identify culture- and context-specific multisectoral approaches, particularly under restricted living conditions due to COVID-19.

## Figures and Tables

**Table 1:** Characteristics of the study population among Thai children aged 12–59 months, 2015–2016.

Variables	Total	Boys	Girls	Variables	Total	Boys	Girls
No. of subjects	9060	4661	4399	Nutritional status			
Child Age (in months)				Stunted (HAZ < −2)	10.8	12.0	9.5
12–23	24.5	26.1	22.9	Wasted (WHZ < −2)	4.9	4.6	5.1
24–35	25.1	24.3	25.9	Overweight (WHZ > 2)	9.2	10.0	8.5
36–47	25.5	25.0	26.0	Concurrent stunting and wasting (%)	0.6	0.8	0.4
48–59	24.9	24.6	25.3	Concurrent stunting and overweight (%)	1.2	1.7	0.6
Birth weight (g)				Health status			
Low (<2500)	8.3	7.7	9.0	Diarrhea (%)	5.3	5.3	5.2
Excess (>4000)	1.8	2.2	1.4	Vaccinations (%)			
Socioeconomic status (SES)				BCG	97.1	96.6	97.7
Residential area (rural, %)	61.4	61.8	60.9	Polio			
Region				1	97.1	96.5	97.7
Bangkok	6.6	7.0	6.3	2	96.3	95.6	97.0
Central	28.5	28.7	28.2	3	94.1	93.9	94.4
North	17.8	17.8	17.7	DPT			
Northeast	31.3	30.3	32.3	1	96.1	95.6	96.6
South	15.9	16.2	15.5	2	94.3	93.7	94.9
Wealth index quintile				3	91.8	91.3	92.3
Poorest	23.6	23.9	23.3	HepB			
Second	22.2	21.8	22.5	At birth	96.7	96.2	97.3
Middle	19.1	19.2	19.1	1	94.8	94.1	95.5
Fourth	20.6	20.8	20.4	2	92.6	91.3	93.9
Richest	14.5	14.3	14.7	3	88.7	87.6	89.8
Mother’s education				Measles (MMR1)	94.5	94.1	94.9
None	4.4	5.3	3.5	Fully vaccinated	84.8	83.4	86.2
Primary	31.4	30.3	32.6	WASH indicator (%)			
Secondary	44.6	45.2	44.0	Use of improved water sources	86.9	86.2	87.6
Higher	19.5	19.2	19.9	Safe disposal of child’s feces	25.9	25.6	26.2
Missing/DK	0.1	0.1	0.0	Place for handwashing	59.3	58.2	60.4

DK, don’t know; HAZ, height-for-age z-score; WHZ, weight-for-height z-score; WASH, water, sanitation, and hygiene; BCG, Bacillus Calmette–Guérin; DPT, diphtheria–pertussis–tetanus; HepB, hepatitis B.

**Table 2:** Multiple logistic regression analyses for the association of nutritional status with full immunization and WASH status among Thai children aged 12–59 months, 2015–2016.

Variables	Stunting			Wasting			Overweight		
	OR	95% CI		OR	95% CI		OR	95% CI	
All ^1^									
Incomplete vaccine coverage	1.47	(1.24–1.75)	***	1.67	(1.31–2.12)	***	1.24	(1.01–1.51)	*
Use of unimproved water sources	0.48	(0.14–1.57)		0.48	(0.08–2.87)		2.43	(1.27–4.64)	**
Unsafe disposal of child’s feces	1.05	(0.87–1.25)		0.90	(0.68–1.18)		0.82	(0.67–1.02)	
Unavailability of handwashing place	0.91	(0.74–1.11)		0.65	(0.46–0.92)	*	1.06	(0.85–1.31)	
Boys ^1^									
Incomplete vaccine coverage	1.54	(1.23–1.93)	**	1.08	(0.75–1.56)		1.34	(1.04–1.73)	*
Use of unimproved water sources	0.16	(0.02–1.59)		0.31	(0.02–6.06)		2.69	(1.27–5.69)	*
Unsafe disposal of child’s feces	0.86	(0.68–1.10)		1.14	(0.77–1.69)		0.89	(0.66–1.19)	
Unavailability of handwashing place	0.85	(0.65–1.11)		0.75	(0.47–1.22)		1.13	(0.86–1.49)	
Girls ^1^									
Incomplete vaccine coverage	1.24	(0.94–1.64)		2.44	(1.75–3.40)	***	1.12	(0.81–1.55)	
Use of unimproved water sources	1.22	(0.29–5.15)		0.98	(0.10–9.53)		1.45	(0.33–6.37)	
Unsafe disposal of child’s feces	1.46	(1.09–1.95)	*	0.71	(0.48–1.07)		0.75	(0.55–1.02)	
Unavailability of handwashing place	0.85	(0.61–1.19)		0.60	(0.36–1.01)		0.98	(0.69–1.39)	

*** *p* < 0.001, ** *p* < 0.01, * *p* < 0.05. After adjustment for birth weight, residential area, region, wealth index, and mother’s education, we examined the associations of nutritional status with health status and WASH status. ^1^ Number of participants included in the analyses: All: 9830 stunted, 8988 wasted, 9303 overweight; Boys: 5029 stunted, 4582 wasted, 4744 overweight; Girls: 4801 stunted, 4406 wasted, 4519 overweight. Wasting defined as (WHZ < −2) or normal (−2 ≤ WHZ ≤ 2); Overweight defined as (WHZ > 2) or normal (−2 ≤ WHZ ≤ 2). OR, odds ratio; CI, confidence interval; WHZ, weight-for-height z-score; PHC, primary health care; WASH, water, sanitation, and hygiene.

## Data Availability

The dataset is available on the following website: https://mics.unicef.org/surveys (accessed on 28 October 2021).
